# Thymoquinone enhances cisplatin-response through direct tumor effects in a syngeneic mouse model of ovarian cancer

**DOI:** 10.1186/s13048-015-0177-8

**Published:** 2015-07-28

**Authors:** Andrew J. Wilson, Jeanette Saskowski, Whitney Barham, Fiona Yull, Dineo Khabele

**Affiliations:** Department of Obstetrics and Gynecology, Division of Gynecologic Oncology, Vanderbilt University School of Medicine, Nashville, TN USA; Department of Cancer Biology, Vanderbilt University Medical Center, Nashville, TN USA; Vanderbilt-Ingram Cancer Center, Vanderbilt University Medical Center, Nashville, TN USA; Department of Obstetrics and Gynecology, Vanderbilt University Medical Center, B1100 Medical Center North, Nashville, TN 37232 USA

**Keywords:** NF-κB activity, Ovarian cancer, Syngeneic mouse model, Macrophages, Thymoquinone, Cisplatin

## Abstract

**Background:**

Ovarian cancer is the most lethal gynecologic malignancy characterized by the frequent development of resistance to platinum chemotherapy. Finding new drug combinations to overcome platinum resistance is a key clinical challenge. Thymoquinone (TQ) is a component of black seed oil that exerts multiple anti-tumorigenic effects on cells, including inhibition of NF-κB and promotion of DNA damage. We aimed to determine whether TQ enhances cisplatin cytotoxicity in cultured ovarian cancer cells and in an established murine syngeneic model of ovarian cancer.

**Methods:**

Ovarian cancer cell viability *in vitro* was measured by sulforhodamine B (SRB) assays, and drug interactions tested for synergism by isobologram analysis. ID8-NGL mouse ovarian cancer cells stably expressing an NF-κB reporter transgene were injected intra-peritoneally into C57BL/6 mice. After 30 day TQ and/or cisplatin treatment, we measured the following indices: tumor burden (ascites volume, number of peritoneal implants and mesenteric tumor mass); NF-κB reporter activity (luciferase assay); protein expression of the double-strand DNA break marker, pH2AX(ser139), the proliferation markers, Ki67/mib-1 and PCNA, and the apoptosis markers, cleaved caspase-3, cleaved PARP and Bax; and mRNA expression of NF-κB targets, TNF-α and IL-1β. Two-tailed Mann–Whitney tests were used for measuring differences between groups in mouse experiments.

**Results:**

In SRB assays, TQ and cisplatin synergized in ID8-NGL cells. In mice, cisplatin significantly reduced cell proliferation and increased apoptosis in tumors, resulting in decreased overall tumor burden. Combining TQ with cisplatin further decreased these indices, indicating co-operative effects between the drugs. TQ treatment promoted cisplatin-induced pH2AX expression in cultured cells and in tumors. While NF-κB inhibition by TQ induced anti-tumor effects *in vitro*, we made the unexpected observation that TQ alone increased both tumor NF-κB activity and formation of ascites *in vivo*.

**Conclusions:**

TQ enhanced cisplatin-mediated cytoxicity in ovarian cancer cells *in vitro* and in a mouse syngeneic model, effects associated with increased DNA damage. However, our results strongly caution that TQ treatment alone may have an overall deleterious effect in the immunocompetent host through stimulation of ascites. Since TQ is a potential candidate for future clinical trials in ovarian cancer patients, this finding has considerable potential relevance to the clinic.

**Electronic supplementary material:**

The online version of this article (doi:10.1186/s13048-015-0177-8) contains supplementary material, which is available to authorized users.

## Background

Ovarian cancer is the most common cause of death from gynecologic malignancies in the United States [1]. Most women with epithelial ovarian cancers are diagnosed with advanced, metastatic disease characterized by widespread peritoneal carcinomatosis and abdominal ascites [2]. The development of resistance to platinum chemotherapy (carboplatin and cisplatin) is common in advanced disease [3]. Therefore, identifying new drugs to improve platinum response is critical to prolonging the life of women with refractory disease.

Thymoquinone (TQ) is a product of the medicinal plant *Nigella sativa* which has promising anti-tumor efficacy in preclinical models of human cancer [4–7]. Multiple molecular mechanisms of action have been described for the demonstrated ability of TQ to reduce tumor growth and survival in these preclinical studies. These include activation of tumor suppressor genes such as PTEN and p21, reducing pro-inflammatory and angiogenic signals via inhibition of NF-κB signaling, an important molecular link between inflammation and cancer [8–13], and induction of DNA damage through generation of reactive oxygen species (ROS) [4–6]. Early clinical trials have shown promising lack of toxic effects in patients with symptoms of cardiovascular disease such as hypertension and hypercholesterolemia [6]. Only one Phase 1 trial has been reported for thymoquinone administration in 21 cancer patients, with no toxic or therapeutic effects detected over treatment times ranging from 1 to 20 weeks [14]. Definitive trials for establishing safe and effective doses of TQ in cancer patients are currently lacking, but are well supported by preclinical data [4–7].

Several mechanisms of resistance to platinum compounds in cancer cells have been identified [15]. First, cisplatin treatment is known to induce NF-κB [16], and NF-κB inhibitors potentiate the anti-tumor activity of various cytotoxic agents [17]. Second, cisplatin induces double-strand DNA breaks by intercalating into DNA [15], and its effects are reduced in ovarian cancer cells with intact DNA repair capacity [18]. We have shown previously that drugs which promote DNA damage or inhibit DNA repair (e.g. histone deacetylase inhibitors) can sensitize ovarian cancer cells to cisplatin and DNA-damaging drugs [19, 20]. Since TQ has multiple cellular effects that could potentiate cisplatin response, we hypothesized that TQ would sensitize ovarian cancer cells cultured *in vitro* and in our syngeneic model to the cytotoxic effects of cisplatin.

Most preclinical models are limited by the fact that drug effects are tested on cancer cells in the absence of the supporting tumor microenvironment, essential for cancer progression in vivo. For this reason, we generated a mouse syngeneic model using ID8 mouse ovarian cancer cells grown intra-peritoneally in C57BL/6 mice [21]. The cells have a stably integrated NF-κB reporter plasmid, allowing for quantification of tumor NF-κB activity in response to drug treatment during intraperitoneal abdominal carcinomatosis accompanied by ascites formation.

In this study, we show that combined TQ and cisplatin treatment induced synergistic anti-tumor effects in cultured ID8-NGL cells, and reduced tumor burden, proliferative and apoptotic markers in ID8-NGL-derived tumors. These combinatorial effects were associated with enhanced expression of the DNA damage marker, pH2AX(ser139), compared to either drug alone. Although TQ-mediated inhibition of NF-κB was observed in vitro, our syngeneic model showed an unexpected increase in tumor NF-κB activity and ascites volume with TQ treatment alone. These results emphasize the potential of targeting DNA damage as a therapeutic approach in ovarian cancer, but also that strongly caution TQ may have an overall deleterious effect through promotion of ascites formation. Since TQ is a likely candidate for future clinical trials in cancer patients [5–7], this finding has considerable potential relevance to the clinic.

## Materials and methods

### Cell culture

Mouse ovarian cancer cells stably expressing a NF-κB reporter plasmid, ID8-NGL [21], were cultured in 10 % FBS-supplemented DMEM High-Glucose medium with 400 μg/ml G418, and passaged by standard techniques. The human ovarian cancer cell lines, OVCAR3 and NCI/ADR-RES were cultured as previously described [22]. Cultured ID8-NGL cells were treated with increasing concentrations of cisplatin (Sigma Chemical Co., Cat# 479306) and/or the NF-κB inhibitor, thymoquinone (TQ; Sigma Chemical Co., Cat# 274666).

### Cell viability assays

Sulforhodamine B (SRB) assays were used to determine cell proliferation and cytotoxicity in response to TQ and/or cisplatin as previously described [22]. Absorbance was measured at 510 nm using a Spectramax M5 spectrophotometer (Molecular Devices, Sunnyvale, CA) in the High-Throughput Screening Core of the Vanderbilt Institute of Chemical Biology. The interaction between fixed ratios of TQ and cisplatin was measured with the Combination Index (CI) method [23]. A CI level of <0.9, CI = 0.9–1.1 and CI > 1.1 indicates synergy, additivity and antagonism respectively, between drug combinations.

### Animal model and drug treatment

Wild-type C57BL/6 mice were injected intra-peritoneally (IP) with 1 × 10^7^ ID8-NGL cells suspended in 200 μl sterile PBS [21]. Thirty days after ID8-NGL injection, mice were randomized into the following treatment groups: vehicle (PBS), TQ (20 mg/kg thrice weekly), cisplatin (2 mg/kg weekly) and the TQ/cisplatin combination via the IP route for 30 days (*n* = 5). No signs of drug toxicity were observed in the single or combination treatment mice. Tumor progression was monitored by body weight and abdominal girth measurements. At time of sacrifice, abdominal ascites fluid was extracted with hypodermic syringe, and volume measured. Tumor implants in the peritoneal wall and the mesentery were measured, then harvested and snap frozen or formalin-fixed for further analysis. The experimental protocol was reviewed and approved by the Institutional Animal Care and Use Committee at Vanderbilt University.

### Luciferase assays

Luciferase activity was measured in harvested tumors following tissue homogenization in 1 ml reporter lysis buffer, and in whole cell protein extracts from cells grown in vitro, using the Promega Luciferase Assay system (Cat#4030). Activity was analyzed using a GloMax Luminometer (Promega, Madison, WI). Results were expressed as relative light units (RLU) normalized for protein content, as measured by the Bradford assay (Bio-Rad, Cat# 500-0002).

### RNA extraction and quantitative RT-PCR (QPCR)

RNA from snap-frozen tumors was isolated using the RNeasy Mini kit (Qiagen, Valencia, CA) and QPCR performed as described [21]. Steady-state mRNA levels of the established NF-κB targets, TNF-α and IL-1β, were expressed relative to corresponding GAPDH levels using the comparative 2^ΔΔCt^ method [24]. Primers sequences used were as previously described [21, 25, 26].

### Immunofluorescence

Processing, embedding and sectioning of formalin-fixed ID8-NGL tumor tissue, and hematoxylin and eosin staining for histology, were performed in The Allergy/Pulmonary & Critical Care Med Division Immunohistochemistry Core at Vanderbilt [27]. Immunofluorescence analysis of formalin-fixed paraffin-embedded tumor tissue or methanol-fixed cultured ID8-NGL cells was performed using standard techniques [21, 19]. The following primary antibodies: mouse monoclonal anti-pH2AX(ser139) (EMD Millipore, Cat# 05-636, 1:250 dilution); rabbit polyclonal anti-Ki67/Mib-1 (Abcam, Cat# ab16667; 1:200 dilution); and rabbit polyclonal anti-cleaved caspase-3 (Cell Signaling Technology, Cat# 9661; 1:100 dilution), were used. Secondary antibody used was goat anti-rabbit Alexa Fluor 488 (Life Technologies, Cat# 11070) (all 1:200 dilution). Images were acquired and analyzed as previously described [21, 19]. For quantifying the percentage of, where applicable, tumor cells or macrophages positive for these proteins, at least 5 independent fields were assessed with at least 200 cells counted per sample.

### Western blotting

In ID8-NGL cells treated with TQ (25 μM) and/or cisplatin (1 μM), or in drug-treated tumors, whole cell protein isolation, subcellular fractionation, western blotting and signal detection were performed as described [28, 29]. Primary antibodies used were rabbit polyclonal anti-PARP (Cell Signaling Technology; Cat# 9542; 1:1000 dilution), mouse monoclonal anti-PCNA (Santa Cruz, Cat# PC10, 1:1000 dilution), rabbit polyclonal anti-Bax (EMD Millipore, Cat# 06-499, 1:1000 dilution), and mouse monoclonal anti-pH2AX(ser139) (EMD Millipore, Cat# 05-636, 1:500 dilution). Mouse monoclonal anti-β-actin (Sigma Chemical Co., Cat# A5441 1:10000 dilution) and rabbit polyclonal anti-H2AX (EMD Millipore, Cat# 06-627, 1:500 dilution) were used as loading control, where appropriate.

### Statistical analysis

Unless otherwise indicated, values shown for *in vitro* experiments were the mean + SE of 3 independent experiments, with comparison of groups performed by 2-tailed Student’s *t* test. Comparison of groups in in vivo experiments was performed by 2-tailed Mann–Whitney test. A *p* value <0.05 is considered statistically significant.

## Results

### TQ enhances anti-tumor effects of cisplatin in cultured ovarian cancer cells

**Fig. 1:**
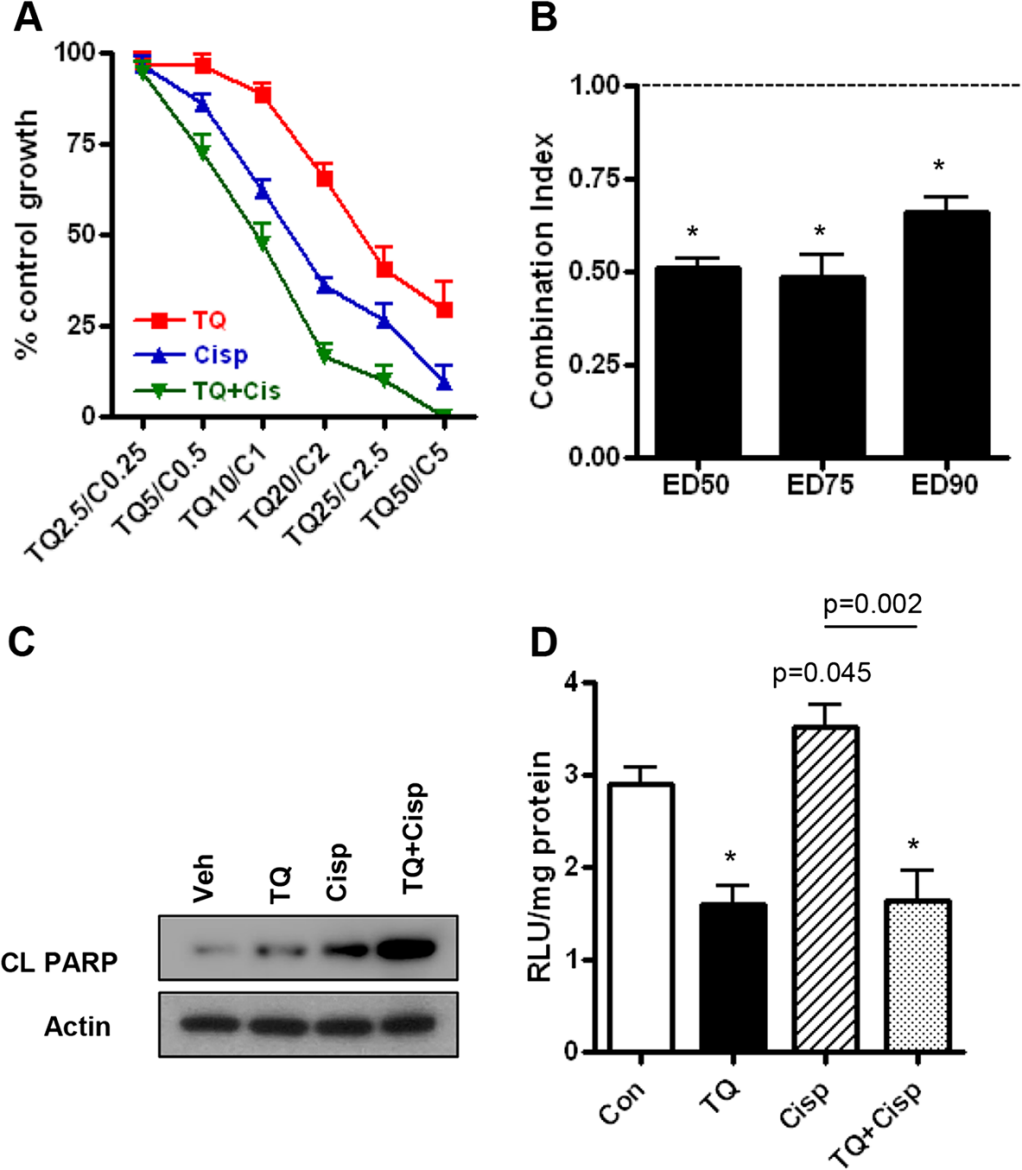
TQ and cisplatin synergistically decrease cell viability in cultured ID8-NGL cells. **a** SRB assay showing effects of TQ and/or cisplatin (concentrations in μM, 72 h) in the mouse ovarian cancer cell line, ID8-NGL, expressed as a percentage of control. **b** Isobologram analysis for ID8-NGL cells showing Combination Index for Effective Doses (ED) ED50, ED75 and ED90. A Combination Index < 1 indicates a synergistic drug interaction between TQ and cisplatin. Values are mean ± SD for 3 experiments; *p < 0.01 below 1, Student’s *t* test. **c** Western blot analysis of cleaved PARP (CL PARP) following 24 h treatment of ID8-NGL cells with TQ (25 μM), cisplatin (1 μM) or the combination. Actin was used as a loading control. **d** Luciferase assay of ID8-NGL cells following 24 h treatment with vehicle, TQ (25 μM), cisplatin (1 μM) or the combination. Values are mean ± SD for 3 experiments; *p < 0.01 compared to control, Student’s *t* test

Here, we aimed to determine whether TQ can sensitize ovarian cancer cells cultured in vitro to the cytotoxic effect of cisplatin. First, the combination of TQ and cisplatin was tested in SRB cell growth and viability assays. In ID8-NGL mouse ovarian cancer cells. the drug combination induced synergistic inhibitory effects on cell viability, as shown by a Combination Index significantly less than 1 at Effective Doses (ED) ED50, ED75 and ED90 (Fig. 1a and b). Furthermore, the pro-apoptotic effects of cisplatin were markedly enhanced by combination TQ treatment (Fig. 1c), indicating that apoptosis was an important mechanism of action of the anti-tumor effects of the combined drugs.

The TQ and cisplatin combination was also tested in the established human ovarian cancer cell lines, OVCAR3 and NCI/ADR-RES, which are relatively resistant to cisplatin [22]. A synergistic decrease in viability of OVCAR3 and NCI/ADR-RES cells was observed with combination treatment with TQ and cisplatin (see Additional file 1: Figure S1).

TQ is known to have a wide range of cellular effects that may contribute to its anti-tumor actions, including inhibition of NF-κB [4, 30]. Cisplatin-induction of NF-κB is an established mechanism of cisplatin-resistance in cancer cells [16]. Therefore, we determined effects of combining TQ and cisplatin on NF-κB activity in ID8-NGL cells. As shown in Fig. 1d, TQ reduced NF-κB reporter activity by approximately 50 % in luciferase assays after treatment for 24 h. Cisplatin induced a modest increase in NF-κB reporter activity (20.1 ± 2.7 %, *p* = 0.045) (Fig. 1d), which was reduced to levels comparable to TQ alone by combining TQ and cisplatin.

### TQ enhances anti-tumor effects of cisplatin in a syngeneic mouse model of ovarian cancer

Based on our in vitro results, we hypothesized that TQ treatment would produce co-operative effects when combined with cisplatin. We have shown in a previous study that TQ treatment for 10 days, starting at day 30 after tumor injection, reduced NF-κB activity as expected in ID8-NGL-derived tumors [21]. However, at this relatively early time point, tumor burden was minimal and was not readily quantifiable. Therefore, we chose to treat mice for 30 days to maximize our indices of tumor burden (ascites volume, number of peritoneal implants and mesenteric tumor mass) at the 60 day study end-point [21].

**Fig. 2:**
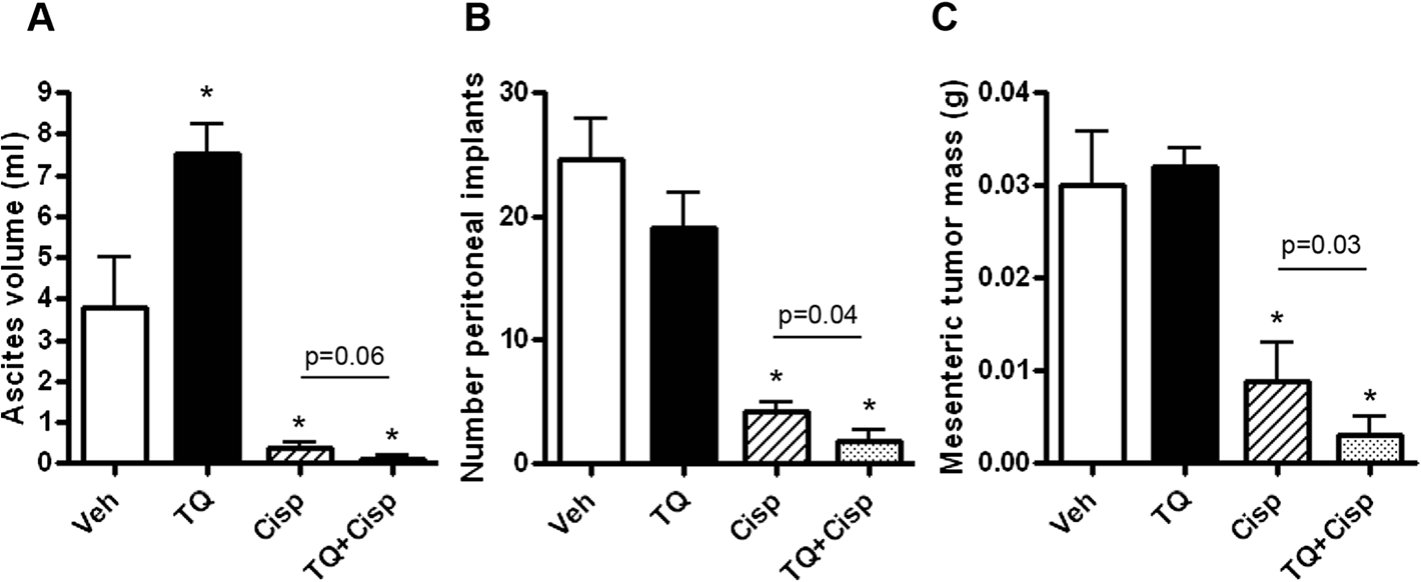
TQ and cisplatin combine to decrease tumor burden. BL/6 mice were injected with ID8-NGL cells. Thirty days after tumor cell injection, mice were randomized and treated with PBS vehicle (Veh), TQ (20 mg/kg thrice weekly), cisplatin (Cisp; 2 mg/kg weekly) or the TQ/cisplatin combination for 30 days and sacrificed. At sacrifice, **a** ascites fluid volume, **b** number of peritoneal implants and **c** mesenteric tumor mass were measured. Values are mean + SD for 5 mice per group; *p < 0.01 relative to vehicle; all *p* values determined were by Mann–Whitney test

Treatment with TQ alone induced a 2-fold increase in ascites volume at day 60 compared to vehicle-treated mice (Fig. 2a). However, there was no overall difference in peritoneal (Fig. 2b) or mesenteric (Fig. 2c) tumors in mice treated with TQ only compared to vehicle. We confirmed these observations for TQ-only treatment in several additional experiments (data not shown). Cisplatin alone reduced all three indices of tumor burden by >80 % compared to vehicle-treated mice. Combining TQ and cisplatin resulted in enhanced reduction in peritoneal implants and mesenteric tumors compared to either drug alone, with a similar effect on ascites volume that just failed to reach statistical significance.

**Fig. 3:**
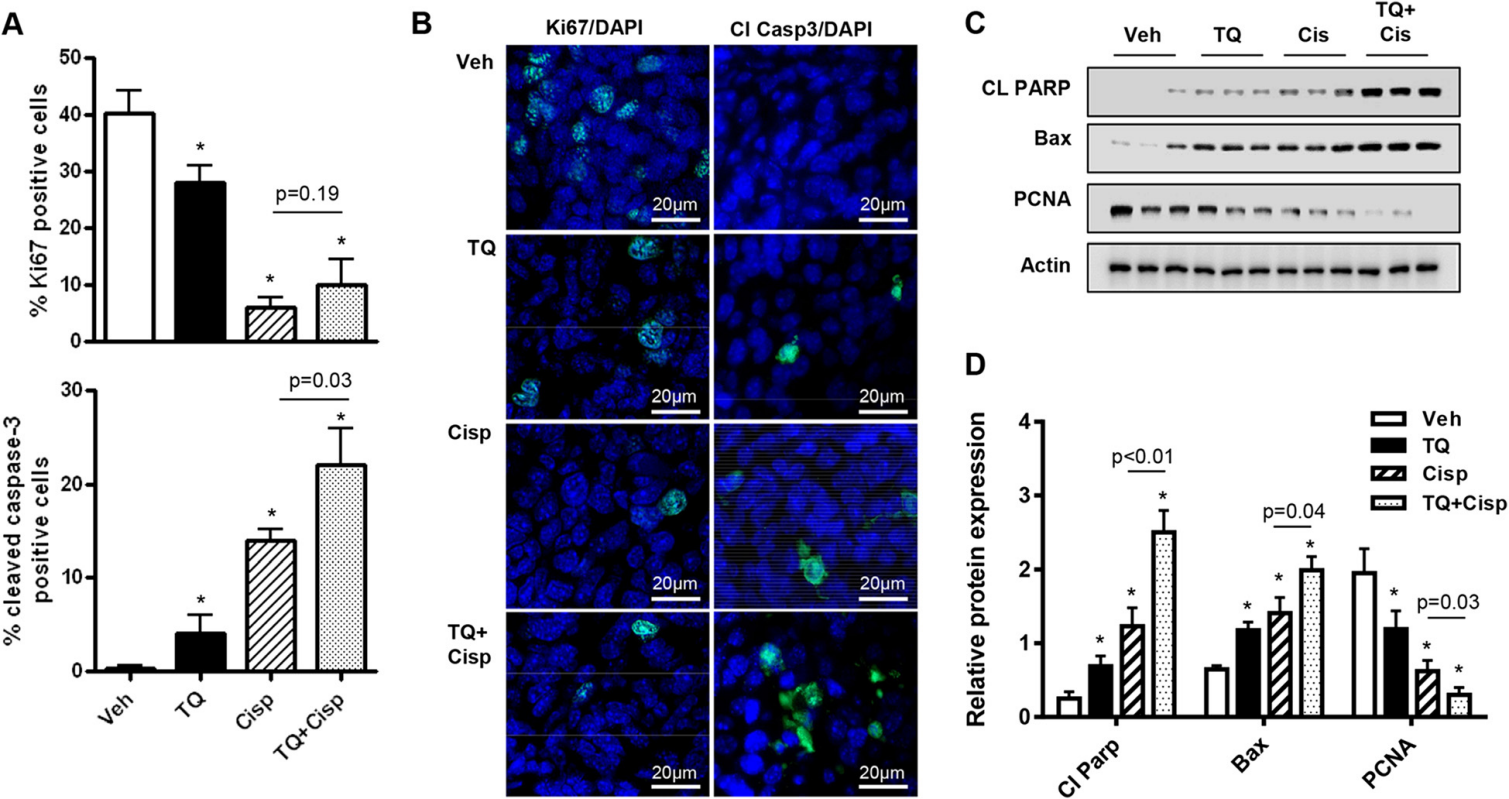
TQ and cisplatin combine to decrease tumor cell proliferation and increase apoptosis. **a** The percentage of tumor cells positive for proliferation (Ki67/mib-1) and apoptosis (cleaved caspase-3) markers in tumors harvested from ID8-NGL-injected mice treated with vehicle, TQ (20 mg/kg), cisplatin (2 mg/kg) or the TQ/cisplatin combination for 30 days. At least 200 cells were counted (×40 objective) for each drug treatment per experiment. **b** Representative fields of drug-treated tumors. Ki67/mib-1 or cleaved caspase-3 are in *green*. DAPI-stained nuclei are in *blue*. **c** Western Blot analysis of cleaved PARP (CL PARP), Bax and PCNA in ID8-NGL tumors harvested from drug-treated mice. Actin was used as a loading control. **d** Densitometry analysis of cleaved PARP, Bax and PCNA expression in tumors relative to corresponding actin levels. Values are mean + SD for 3–5 mice per group; *p < 0.01 relative to vehicle; all *p* values determined by Mann–Whitney test

We next determined drug effects on cell proliferation and apoptosis in the tumors. Immunofluorescence analysis of tumors demonstrated that cisplatin reduced the percentage of cells positive for the proliferation marker, Ki67/mib-1 and increased cells showing expression of the apoptosis marker, cleaved caspase-3 (Fig. 3a and b), consistent with effects on tumor burden. Compared to cisplatin alone, the TQ/cisplatin combination group displayed significantly greater levels of apoptosis (Fig. 3a and b). We then independently validated our results by western blot analysis of two additional markers of apoptosis, cleaved PARP and Bax. As shown in Fig. 3c and d, both TQ and cisplatin significantly increased expression of cleaved PARP and Bax, consistent with their effects on cleaved caspase-3 expression. Moreover, combining the drugs significantly increased expression of cleaved PARP and Bax above that of cisplatin alone (Fig. 3c and d). Similarly, expression of PCNA was significantly reduced by TQ and cisplatin alone, and the TQ/cisplatin combination induced significantly greater effects than cisplatin alone (Fig. 3c and d). Therefore, we showed the anti-tumor effects of the TQ/cisplatin combination involved both enhanced cell apoptosis and reduced cell proliferation.

**Fig. 4:**
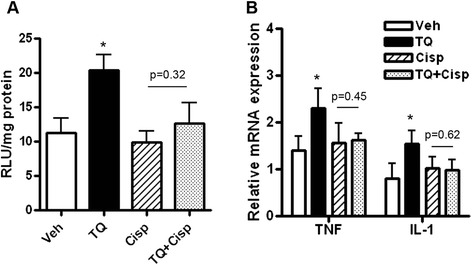
NF-κB activity in ID8-NGL tumors is not inhibited by treatment with TQ and/or cisplatin. BL/6 mice were injected with ID8-NGL cells and treated with vehicle, TQ (20 mg/kg), cisplatin (2 mg/kg) or the combination for 30 days. **a** Luciferase assays of NF-κB reporter activity in harvested ID8-NGL tumors. **b** QPCR analysis of the established NF-κB target genes, TNF-α and IL-1β, in harvested tumors. Relative expression was calculated using corresponding GAPDH levels as the internal control. Values are mean + SD for 5 mice per group; *p < 0.01 relative to vehicle; all *p* values determined by Mann–Whitney test

Contrasting drug effects on NF-κB reporter activity were observed in harvested ID8-NGL tumors compared to cultured cells. As shown in Fig. 4a, treatment with TQ for 30 days unexpectedly led to an overall increase in NF-κB reporter activity in luciferase assays. However, combining TQ and cisplatin abrogated TQ-stimulation, leading to overall similar levels compared to vehicle and cisplatin alone (Fig. 4a). Supporting our results, QPCR analysis of steady-state mRNA levels of the established NF-κB targets, TNF-α and IL-1β, in drug-treated tumors revealed a similar pattern of effect to the luciferase assays (Fig. 4b).

### TQ enhances cisplatin-induced DNA damage in ovarian cancer cells

Results from our in vivo experiments clearly indicate that TQ-mediated inhibition of NF-κB activity was not a mechanism underlying the co-operative effects of the TQ/cisplatin combination. Because of the pronounced increase in, particularly, apoptosis mediated by the combination in both cultured cells and in tumors, we explored alternative mechanisms underlying this pro-apoptotic effect. Since TQ is known to induce DNA damage in cancer cells [31, 32], and sustained DNA damage is linked to apoptosis [33], we hypothesized that TQ enhanced cisplatin-induced DNA damage in cultured ID8-NGL cells and in ID8-NGL-derived tumors in vivo. We identified persistent pH2AX(ser139) activation, which is associated with DNA damage-induced apoptosis [33] and is a sensitive surrogate marker of cytotoxicity in ovarian cancer cells [19, 20].

**Fig. 5:**
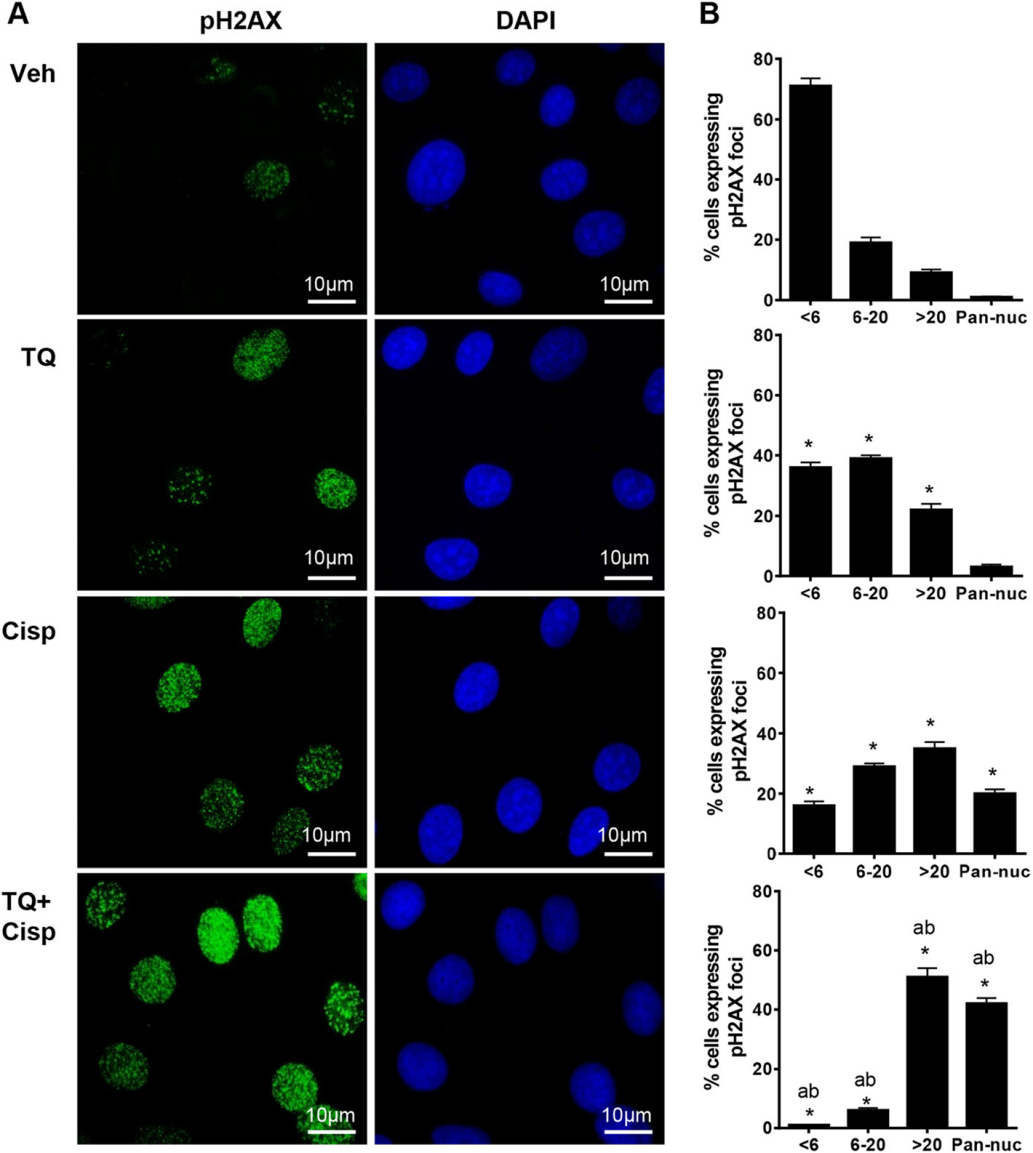
TQ combined with cisplatin increases expression of the DNA damage mark pH2AX(ser139) in cultured ID8-NGL cells. **a** Cultured ID8-NGL cells were treated with vehicle, TQ (25 μM), cisplatin (1 μM) or the combination for 24 h. Immunofluorescent nuclear stain for pH2AX(ser139) is shown in *green*; DAPI-stained nuclei are in *blue*. **b** The number of cell nuclei displaying less than 6 foci (negative), between 6 and 20 foci, greater than 20 foci and diffuse pan-nuclear (Pan-nuc) staining for pH2AX(ser139) foci was quantified and is presented in percentages. At least 200 cells were counted (×60 objective) for each drug treatment per experiment. Values are mean + SD for 3 independent experiments; *p < 0.01 compared to vehicle; ^a^ *p* < 0.01 relative to TQ alone; ^b^ *p* < 0.01 relative to cisplatin alone, all Student’s *t* test

In cultured ID8-NGL cells, we determined patterns of pH2AX(ser139) staining by immunofluorescence analysis following 24 h drug treatment [19, 20] (Fig. 5). Compared to vehicle-treated cells, TQ significantly decreased the number of pH2AX(ser139)-negative cells (<6 foci), and increased the number of cells displaying both intermediate (6–20 foci) and high (>20 foci) pH2AX(ser139) expression. Cisplatin treatment alone had similar effects, with the exception that the proportion of cells displaying pan-nuclear staining (no discrete, countable foci) was also significantly increased compared to controls. Compared to either drug alone, combination treatment further reduced the number of pH2AX-negative cells, and the proportion of cells showing intermediate, high and pan-nuclear pH2AX(ser139) expression.

**Fig. 6:**
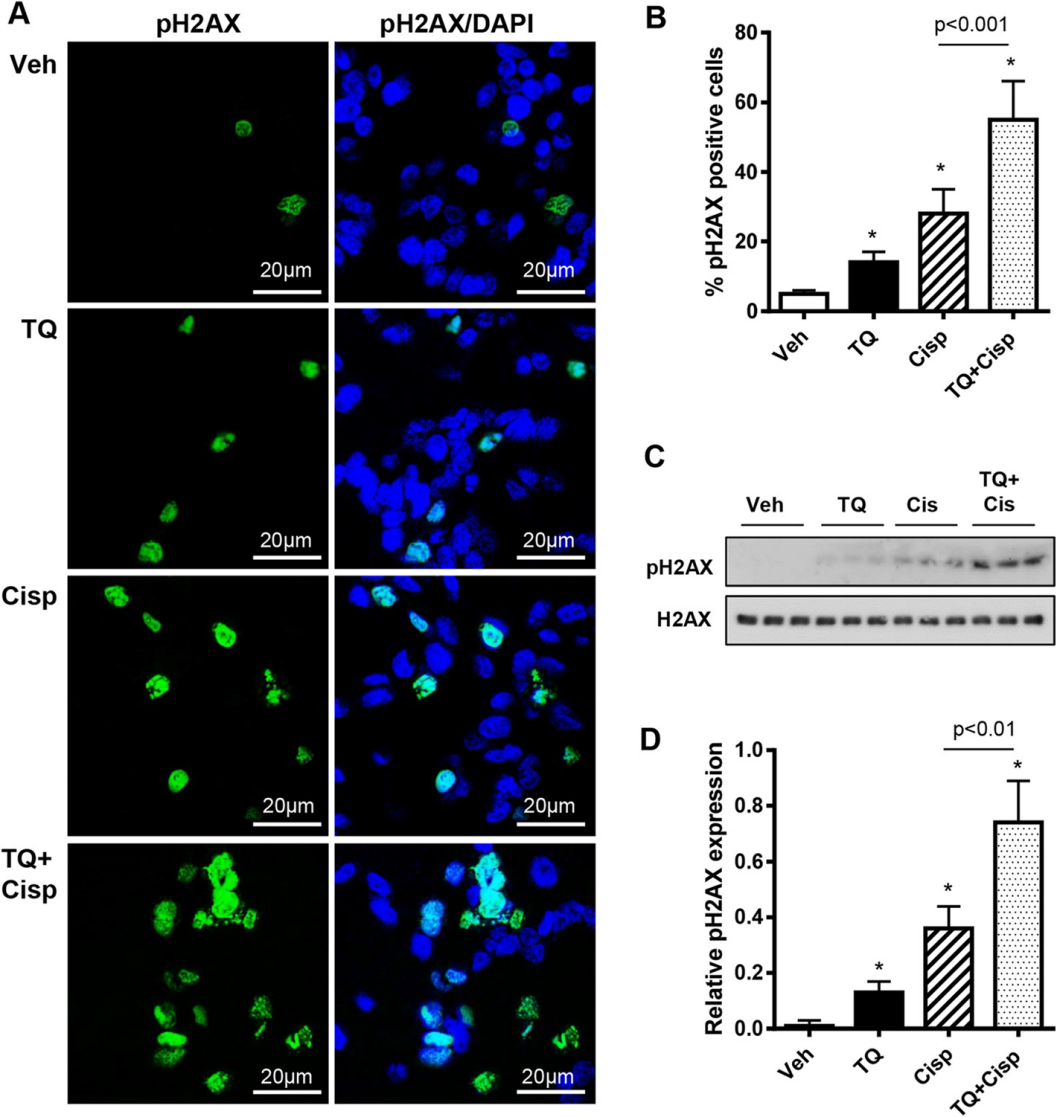
TQ/cisplatin combination increases pH2AX(ser139) expression in ID8-NGL-derived tumors. **a** Representative fields showing pH2AX(ser139) staining (*green*) and DAPI-stained nuclei (*blue*) in tumors harvested from ID8-NGL-injected mice treated with vehicle, TQ (20 mg/kg), cisplatin (2 mg/kg) or the TQ/cisplatin combination for 30 days. **b** The percentage of tumor cells positive for pH2AX(ser139) in drug-treated tumors. At least 200 cells were counted (×40 objective) for each drug treatment. **c** Western Blot analysis of pH2AX(ser139) expression in tumors. Total H2AX was used as a loading control. **d** Densitometry analysis of pH2AX(ser139) expression in tumors relative to corresponding H2AX levels. Values are mean + SD for 3–5 mice per group; *p < 0.01 relative to vehicle; all *p* values determined by Mann–Whitney test

We found a similar pattern of effect in drug-treated tumors, where the highest percentage of tumor cells positive for pH2AX(ser139) in immunofluorescence assays was observed following TQ and cisplatin combination treatment, significantly higher than for cisplatin alone (Fig. 6a and b). We obtained similar results when pH2AX(ser139) expression was measured by western blot (Fig. 6c and d).

## Discussion

Platinum resistance is common in ovarian cancer, and identifying novel drug combinations to enhance efficacy of platinum drugs such as carboplatin and cisplatin is a promising strategy. In this study, we tested the ability of TQ to sensitize ovarian cancer cells to cisplatin treatment in established preclinical models. First, we demonstrated that the combination of TQ and cisplatin had synergistic inhibitory effects on cell viability and survival in cultured human and mouse ovarian cancer cells. Second, compared to either drug alone, there were enhanced effects of the combination on established indices of tumor burden (peritoneal implants and mesenteric tumor mass), proliferation and apoptosis in a syngeneic mouse ovarian cancer model [21].

TQ is known to have a myriad of cellular effects in tumor cells, including promotion of DNA damage through generation of reactive oxygen species and inhibition of NF-κB activity [4–6]. Our results indicate that TQ was able to enhance DNA damage induced by cisplatin in both cultured cells and in tumors, consistent with previous studies from this laboratory showing that drugs which promote DNA damage sensitize ovarian cancer cells to the cisplatin-mediated cytotoxicity [19, 20]. In contrast, TQ-mediated inhibition of NF-κB activity could not explain the enhancement of the cisplatin response in cultured cells and tumors. First, cisplatin only weakly induced NF-κB activity in vitro, an established mechanism of cisplatin-resistance [16]. Second, in contrast to cultured cells in vitro, TQ induced tumor NF-κB reporter activity and mRNA levels of the key NF-κB targets, IL-1β and TNF-α, in drug-treated mice.

We acknowledge that the tumor microenvironment could impact drug response in our syngeneic model. For example, it is likely that the unexpected increase in ascites formation and activity of the NGL NF-κB reporter in tumors following the 30-day TQ treatment was mediated through drug effects on the tumor microenvironment. We have previously shown that TQ treatment for 10 days effectively reduces NGL reporter activity within tumors *in vivo* [21]. Furthermore, thrice-weekly TQ treatment results in persistent inhibition of NF-κB activity in cultured ID8-NGL cells in vitro for up to 21 days (unpublished observations). Identifying drug-induced alterations in specific inflammatory cell populations such as macrophages and dendritic cells, and possibly related mechanisms by which prolonged TQ exposure can induce NF-κB activity and ascites formation, will be the focus of future studies.

A possible microenvironmental role for TQ-induction of NF-κB activity and ascites following longer periods of treatment is supported by a recent study showing that the proteasome inhibitor, bortezomib, induces pro-inflammatory effects on the tumor microenvironment, particularly macrophages, subsequently promoting tumor progression in a mouse lung cancer model [34]. Short-term exposure to bortezomib produces opposing effects resulting in inhibition of tumor cell growth [34], similar to the anti-tumorigenic properties of 10 day TQ treatment [21]. It is not known whether the deleterious effects of long-term TQ and bortezomib, both relatively non-specific inhibitors of NF-κB with additional cellular effects [4–6, 34], are due to their actions on NF-κB activity in these preclinical models. However, because of the interest in using systemic NF-κB inhibitors as mono-therapy or in combination with other chemotherapeutic drugs in clinical trials in ovarian cancer patients [35], possible side effects that limit drug efficacy, promote toxicity or have frankly deleterious effects are highly relevant clinically.

In conclusion, our results emphasize the strong potential of targeting DNA damage as a therapeutic approach to improve cisplatin response in ovarian cancer. However, the choice of drug to combine with cisplatin is critical, since we have shown that TQ, a very promising therapeutic agent in preclinical cancer models, had an unexpected deleterious effect through promotion of ascites formation in a syngeneic model. Since TQ is a potential candidate for future clinical trials in cancer patients [5–7], this finding provides caution for its future use in patients with ovarian cancer.

## Additional file

Additional file 1: Figure S1. Isobologram analysis of combination effects of TQ and cisplatin in human ovarian cancer cells *in vitro.*

## Abbreviations

IL: Interleukin
NF-κB: Nuclear factor-kappaB
NGL: NF-κB-GFP-Luciferase
TQ: Thymoquinone
