# Supplementary material for: Thymoquinone enhances cisplatin-response through direct tumor effects in a syngeneic mouse model of ovarian cancer
Source: J Ovarian Res. 2015 Jul 28;8:46. doi: 10.1186/s13048-015-0177-8 (PMC4517540; doi:10.1186/s13048-015-0177-8)
Supplement: Additional file 1: Figure S1. — Isobologram analysis of combination effects of TQ and cisplatin in human ovarian cancer cells in vitro. [file 13048_2015_177_MOESM1_ESM.pdf]

Figure S1

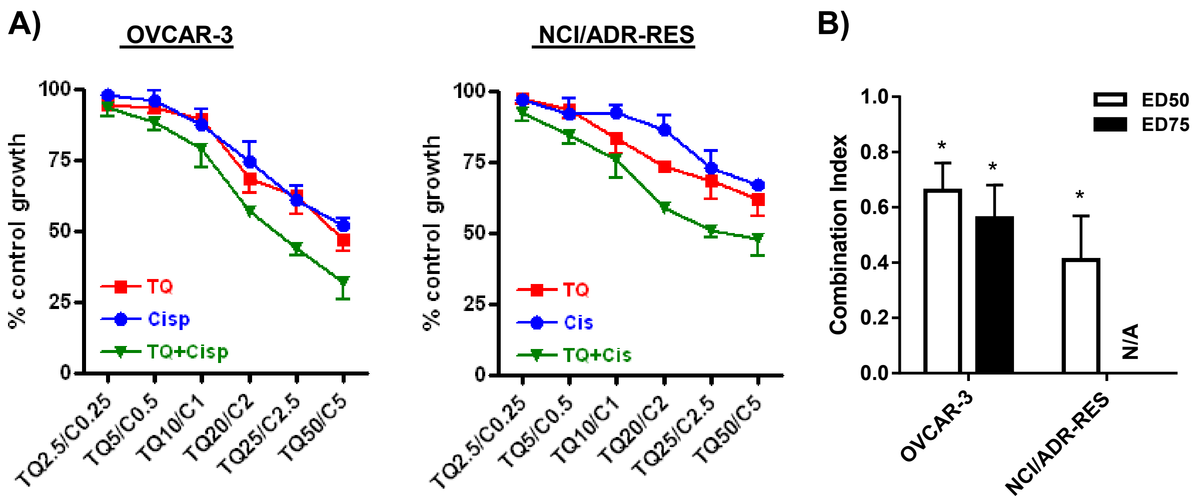

A) SRB assays showing drug effects of TQ and/or cisplatin (concentrations in µM, 72 hours) in 2 human ovarian cancer cell lines (NCI/ADR-RES and OVCAR-3). **B)** Isobologram analysis Combination Index for, where applicable, Effective Doses (ED) ED50,ED75 and ED90. A combination < 1 indicates a synergistic drug interaction between TQ and cisplatin. Values are mean ± SE for 3 experiments; * $p < 0.01$, Student's t test. N/A; not applicable.
